# *n*PoRe: *n*-polymer realigner for improved pileup-based variant calling

**DOI:** 10.1186/s12859-023-05193-4

**Published:** 2023-03-16

**Authors:** Tim Dunn, David Blaauw, Reetuparna Das, Satish Narayanasamy

**Affiliations:** University of Michigan, Ann Arbor, USA

**Keywords:** Germline variant calling, Alignment, N-polymer, Homopolymer, Short tandem repeat, Copy number, Nanopore sequencing, Variable gap penalty

## Abstract

Despite recent improvements in nanopore basecalling accuracy, germline variant calling of small insertions and deletions (INDELs) remains poor. Although precision and recall for single nucleotide polymorphisms (SNPs) now exceeds 99.5%, INDEL recall remains below 80% for standard R9.4.1 flow cells. We show that read phasing and realignment can recover a significant portion of false negative INDELs. In particular, we extend Needleman-Wunsch affine gap alignment by introducing new gap penalties for more accurately aligning repeated *n*-polymer sequences such as homopolymers ($n=1$) and tandem repeats ($2 \le n \le 6$). At the same precision, haplotype phasing improves INDEL recall from 63.76 to $70.66\%$ and nPoRe realignment improves it further to $73.04\%$.

## Background

### Nanopore variant calling

As long read technologies have matured and basecalling accuracy has increased to over $99\%$, their popularity has grown accordingly [1, 2]. Long reads are essential for spanning repetitive regions and unambiguously mapping reads. Last year, the first gapless human genome sequence was constructed by the T2T consortium by combining PacBio HiFi and ONT nanopore long reads [3]. Nanopore sequencing in particular has gained popularity due to its impressive read lengths, low cost, real-time results, and direct calling of base modifications [4–6].

The two current leading nanopore variant callers are Clair3 (developed by the HKUCS Bioinformatics Algorithm Lab) and PEPPER-Margin-DeepVariant (a collaboration between UCSC and Google Health, hereafter referred to as PEPPER) [7, 8]. Both tools have converged on a similar variant calling pipeline: basecalling, read alignment, pileup-based variant calling (using pileup summary statistics), read phasing, and full-alignment variant calling (using all read information).

Despite posting impressive F1 scores ($\ge 0.995$) for SNP calling, nanopore variant callers struggle with accurately identifying INDELs in low-complexity regions [7–9]. Most recent nanopore variant calling advances in this area have come from improvements in machine learning and data representation. For example, the move from prior work Clairvoyante [10] to Clair [9] involved *“an entirely different network architecture and learning tasks”*. Clair3 then split the model into a pileup caller to filter out the noise and a higher-dimensional full-alignment caller to make the more difficult decisions [7]. PEPPER examined sorting reads by haplotype and a new architecture, and DeepVariant explored numerous possible data representations for final calling [8, 11]. Orthogonally, we show that improved INDEL calling performance can be achieved through better read alignment by introducing novel gap penalties for homopolymers and tandem repeats, or “*n*-polymers”.

**Fig. 1:**
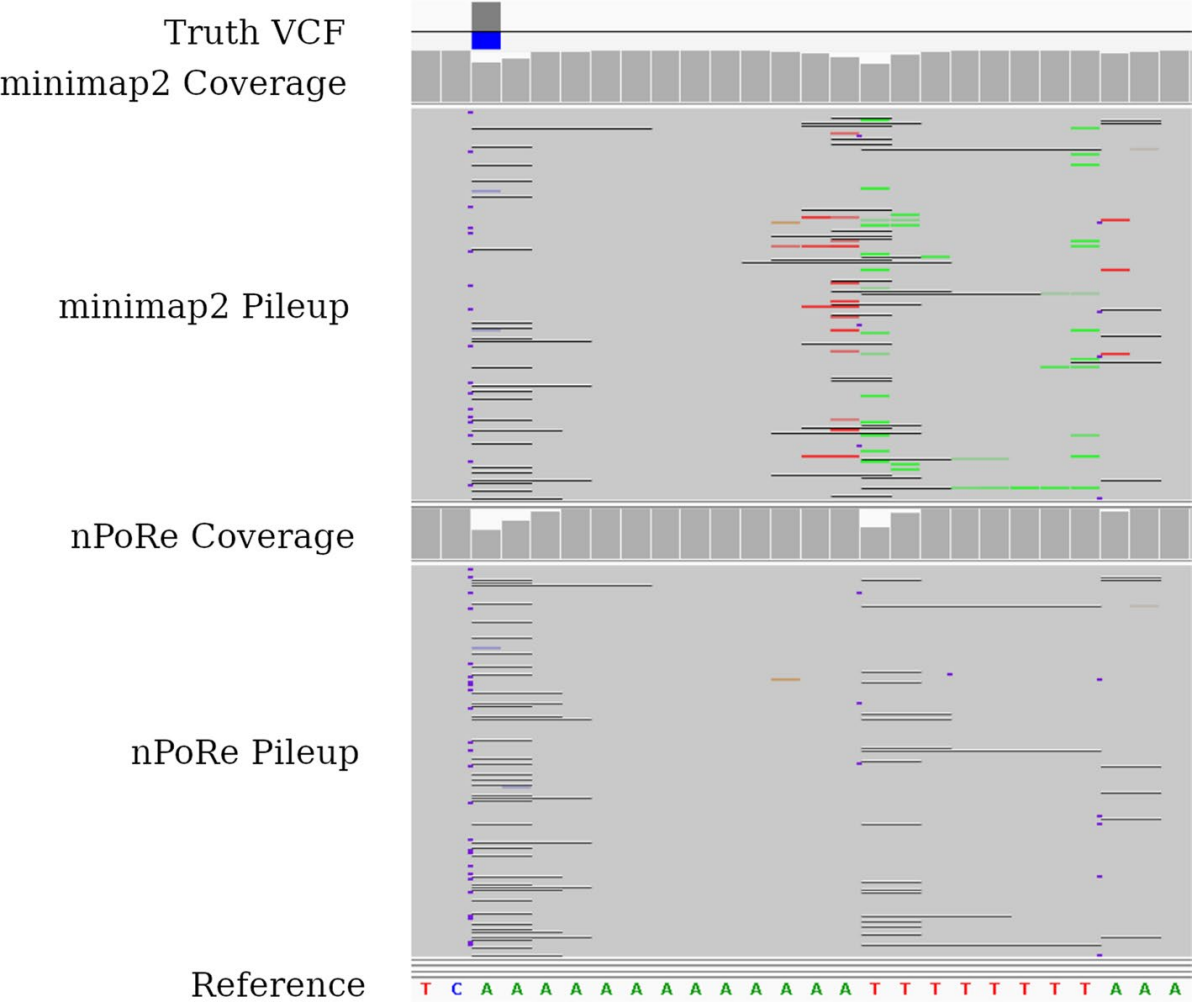
The same reads, aligned by minimap2 and nPoRe, viewed in IGV [12]. Colored lines represent substitutions, black lines represent deletions, and purple vertical bars indicate insertions. Note that nPoRe alignments contain more INDELs than substitutions, and the starts of these INDELs are more consistently placed

### Nanopore read alignment

In order to maximize the accuracy of pileup-based variant calling, reads should be aligned such that actual mutations are always aligned to the same location, despite sequencing errors. We find that simply using traditional affine gap penalties is not ideal because gap penalties $G_{\textrm{open}}$ and $G_{\textrm{extend}}$ are static, regardless of context [13]. For example, although our dataset consisted of only $0.8\%$ INDEL errors, homopolymers of length 10 contained an INDEL error $41.8\%$ of the time. Without lowering the INDEL penalty in the context of repetitive sequences, there is a mismatch between the likelihood and alignment penalty of common sequencing errors. This has an outsized impact on fine-grained read alignment, often at the expense of consistently aligning actual mutations.

Figure 1 demonstrates a specific example where static INDEL gap costs cause poor alignment concordancy in low-complexity regions. Reads are identical in the two pileups shown; only the alignments differ. In this example, two adjacent homopolymers are basecalled with inconsistent lengths. nPoRe recognizes that these two events were most likely independent, and separates them into two homopolymer length mis-calls/variants. In contrast, minimap2 merges two INDELs whenever possible, or aligns homopolymer length differences as SNPs when one homopolymer is lengthened and the other is shortened, resulting in inconsistent alignment. According to the truth VCF, the first homopolymer of all As had a single deletion. Looking at the third base in the coverage graphs in Fig. 1, we can see that nPoRe placed a deletion here for a much larger fraction of reads than minimap2.

**Fig. 2:**
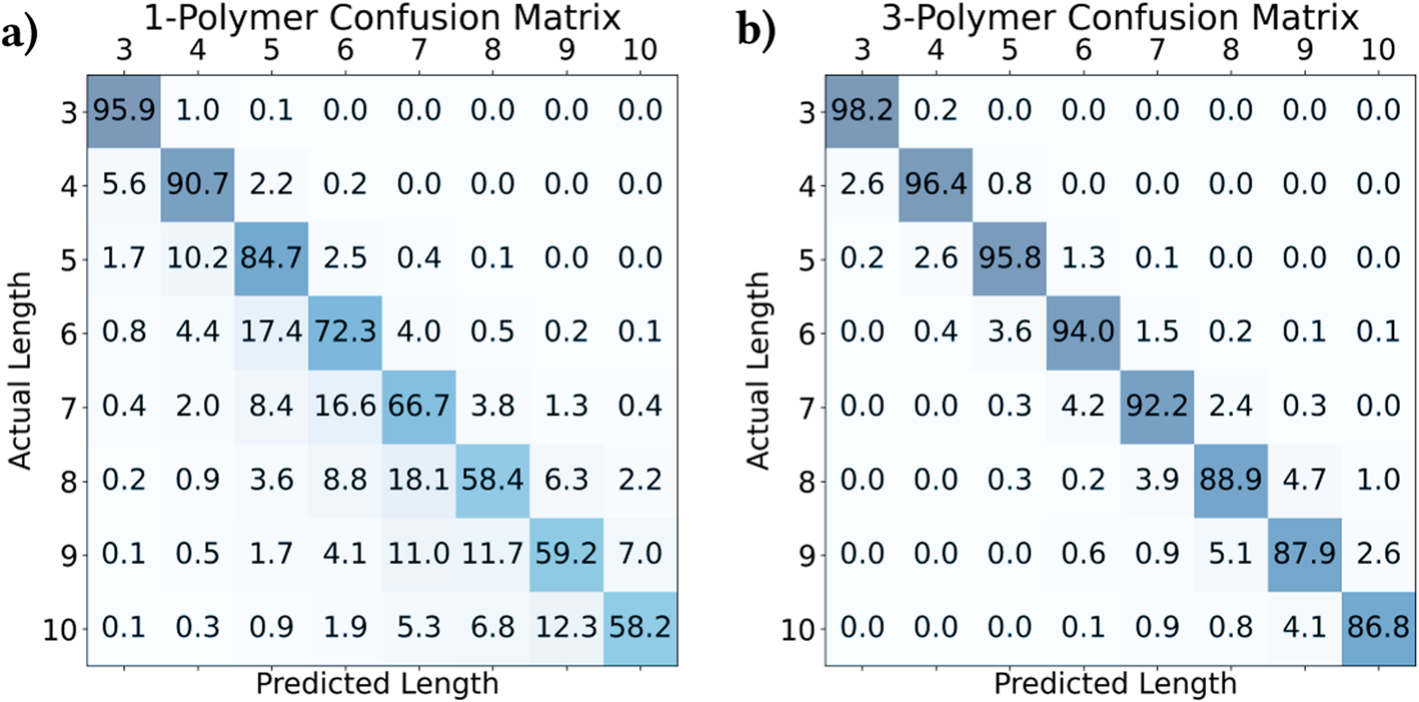
**a** 1-polymer and **b** 3-polymer confusion matrices of actual and predicted *n*-polymer lengths (in percent, by row). “Actual” *n*-polymer lengths are the corresponding reference *n*-polymer lengths to which a read is aligned, and “predicted” *n*-polymer lengths are each read’s basecalled *n*-polymer length

The likelihood of incorrectly basecalling an INDEL within a homopolymer increases significantly as homopolymer length increases. Figure 2a shows the confusion matrix for actual and basecalled homopolymer lengths in our dataset. This same trend is visible for tandem repeats of longer length, though to a lesser extent (Fig. 2b).

### Nanopore INDEL accuracy

Nanopore-based variant callers have historically struggled with INDELs, particularly with recall. Although Clair3 achieves $99.67\%$ precision and $99.60\%$ recall for SNP variant calling, it achieves only $90.86\%$ precision and $64.73\%$ recall for INDELs [7]. PEPPER v4 performs similarly, with $99.61\%$ and $99.62\%$ SNP precision and recall but just over $90\%$ precision and $60\%$ recall for INDELs [8]. The most recent evaluation available shows PEPPER v7 achieving $93\%$ precision and $76\%$ recall for INDELs, at $85\times$ coverage [14].

**Fig. 3:**
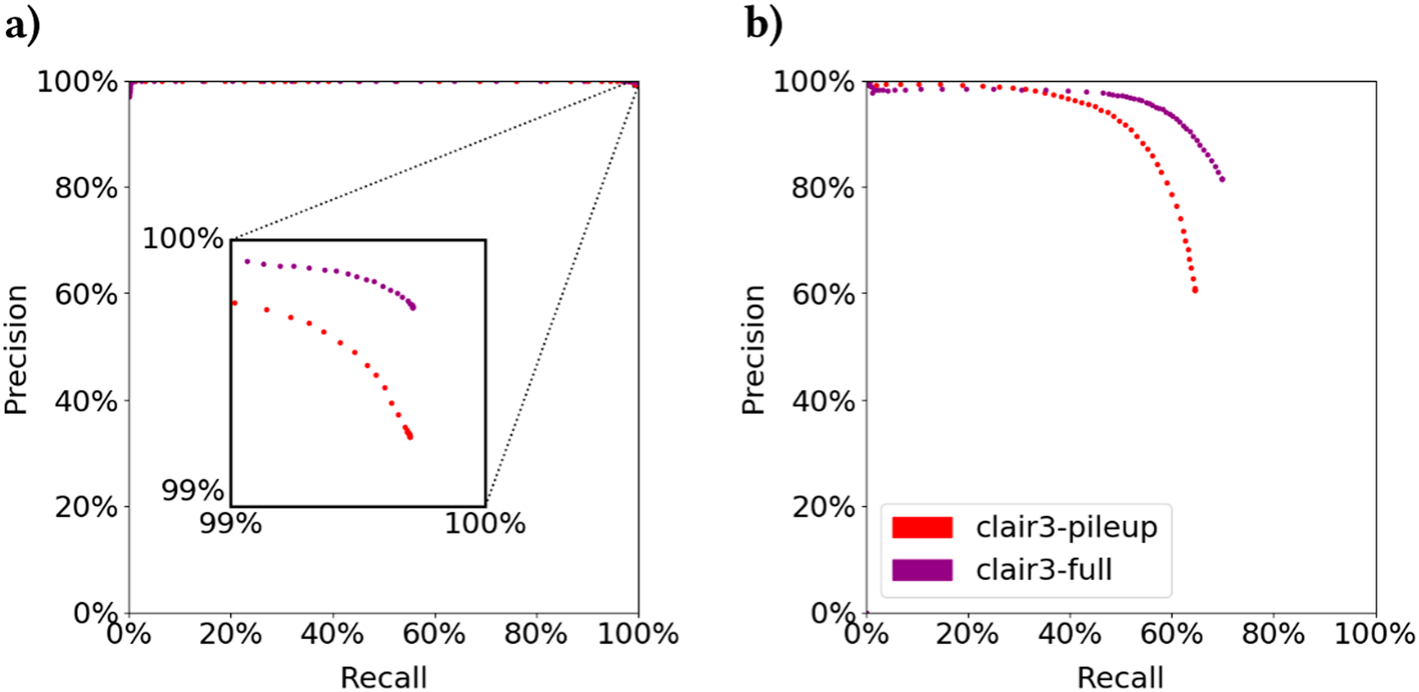
**a** SNP and **b** INDEL germline small variant calling accuracy of baseline clair3-pileup and clair3-full on chr20-22 of GM24385

Our own evaluation confirms these findings, and furthermore attributes the loss of INDEL recall to the first pileup-based variant calling step. Figure 3a and b show SNP and INDEL precision recall curves, respectively, for both Clair3’s pileup and full-alignment models. Note that although the more complex full-alignment model significantly improves precision, it cannot improve recall as dramatically; only variant calls and low-confidence reference calls from the previous pileup-based step are considered.

**Fig. 4:**
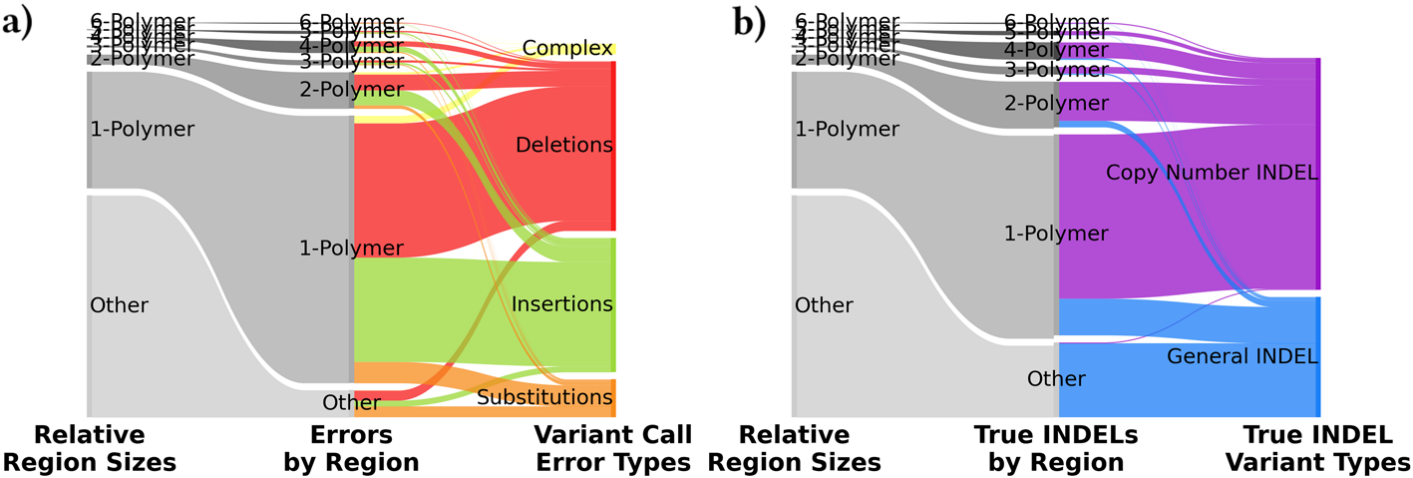
Sankey diagrams demonstrating **a** proportion of pileup-based variant calling errors and **b** true INDEL variants contained within designated *n*-polymer regions of chr20-22 in GM24385

Although substitutions comprise a majority ($83.75\%$) of the actual small germline variants in our dataset, INDELs account for $92.36\%$ of the pileup-based false negative and $80.79\%$ of false positive errors. Figure 4a shows that of these errors, $92.29\%$ occur within *n*-polymer regions, despite *n*-polymer regions covering just $37.07\%$ of evaluated regions. By improving the alignment of reads in these small *n*-polymer regions, we can have a significant impact on overall variant calling accuracy.

Ground truth INDEL mutations are over-represented in *n*-polymer regions as well ($79.64\%$ of all INDELs). This is because Short Tandem Repeat (STR) variation is a common form of mutation due to strand slippage during DNA replication, resulting in one or more copies of a repeated unit being gained or lost. We define copy number INDELs as *n*-polymers (3+ exact copies of the same repeat unit), with a differing number of copies from the expected reference. For example, AAAA$\rightarrow$AAAAA and ATATAT$\rightarrow$ATAT meet this definition, but ATAT$\rightarrow$ATATAT, AATAATAAAT$\rightarrow$AATAAT, and ATATAT$\rightarrow$ATATA do not. Despite our relatively strict definition of *n*-polymer copy number INDELs, however, $65.82\%$ of all INDELs met this classification (Fig. 4b). nPoRe’s algorithm is directly designed to reduce alignment penalties for *n*-polymer copy number INDELs and improve alignment in low-complexity regions.

### Related work

Variable gap penalties have been around for a long time. In 1995, Thompson first introduced per-position gap opening and extension penalties [15]. Since then, the sub-field of homologous protein sequence alignment has made extensive use of variable gap penalties (PIMA [16], FUGUE [17], and STRALIGN [18]) due to a high correlation between INDEL likelihood and the existence of protein secondary structures such as $\alpha$-helices and $\beta$-strands. SSALN was the first to use empirically-determined penalty scores (an approach similar to our own) [19], and SALIGN greatly increased the flexibility of the gap penalty function, although with a corresponding increase in computation [20]. MarginAlign similarly used expectation maximization to obtain robust maximum-likelihood estimates for substitution, insertion, and deletion error rates, and then realigned reads for more accurate single-nucleotide variant calling [21]. Unfortunately, none of the numerous extensions these earlier works made to traditional Needleman-Wunsch alignment are directly applicable to the observed problem of long read *n*-polymer alignment.

Affine gap penalties belong to a larger class of “convex” gap penalties, which also includes piecewise linear and logarithmic gap penalties [22]. These more complex alternatives solve a different problem: reliably grouping several medium-sized gaps into one larger gap. They do this by decreasing the penalty for gap extension with the length of the gap, and are commonly used for accurate alignment of large structural variants [23]. Such convex gap penalties do not solve the issue of fine-grained read alignment because they are still context-agnostic and at short INDEL lengths are highly similar to an affine gap penalty.

One known strategy to mitigate the effect of homopolymer length basecalling errors is “homopolymer compression”, in which repeated bases in a sequence are collapsed (GAAATCCT$\rightarrow$GATCT) [24]. This method is commonly used by graph-based de novo assemblers in the earlier stages of graph construction to improve overlap detection between reads [3, 25, 26]. The recently-developed Verkko assembler goes even further, and compresses *n*-polymers (ATCATCATC$\rightarrow$ATC) [26]. Although *n*-polymer compression is useful for building a consensus graph, the original reads are generally used to generate the final sequence [25, 26]. Read alignment following *n*-polymer compression is equivalent to running nPoRe with a null matrix *N* for *n*-polymer shortening and lengthening penalties. By defining a non-zero matrix *N*, this work penalizes copy number changes according to their measured likelihood.

Several existing works focus on the alignment of Short Tandem Repeats (STRs), although most function as INDEL variant callers rather than read realigners [27–29]. More recently, machine learning based approaches for final variant calling have outperformed these earlier statistical approaches [8, 10, 11, 30]. Several newer works do focus on read realignment, however. ReviSTER is one such tool for revising mis-aligned/mapped reads through reference reconstruction with local assembly, though this is primarily helpful for improving mapping, not alignment. The Broad Institute has incorporated into their standardized analysis pipeline (Genome Analysis ToolKit, or “GATK”) an IndelRealigner, recognizing that INDELs are frequently mis-called as SNPs at read edges [31]. STR-realigner is most similar to our work. It flags STR regions and aligns them separately, allowing repeated traversal of STRs during alignment [32]. They find that this approach improves the consistency of read alignment in and near repeated regions, improving downstream variant calling. STR-realigner was designed for short reads, however, and its runtime $\Omega (n^2)$ which is perfectly fine for short reads of length 101bp is not unacceptable for long reads which regularly reach lengths upwards of 100kbp.

**Fig. 5:**
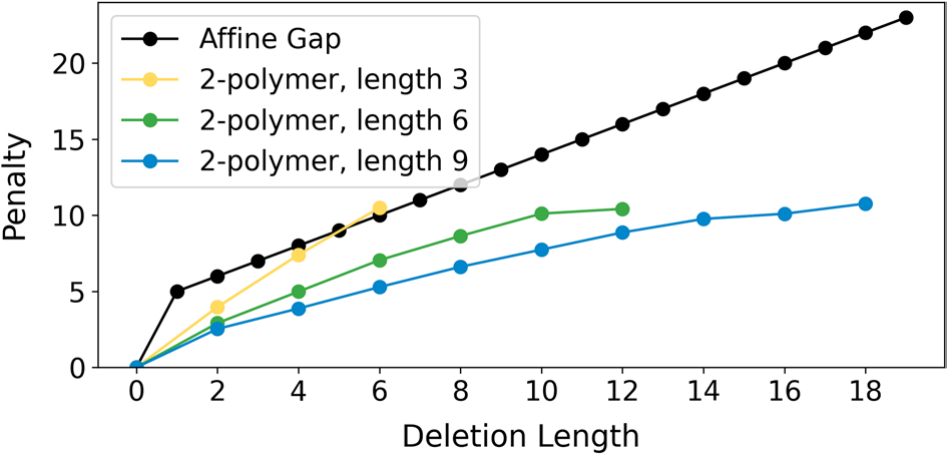
Gap penalties for various copy number deletions, compared to a static affine gap penalty. The penalty is dependent on the local repeat pattern’s periodicity ($n=2$) and length ($l=3,6,9$)

Our work introduces a variable gap penalty for *n*-polymer copy number INDELs, as shown in Fig. 5. INDELs are more likely to occur in *n*-polymers, and so we provide a lower context-specific gap penalty, allowing only copy number INDELs. The exact sequence is not considered in this work; all 2-polymers of length 3 are scored the same (e.g. ATATAT and TGTGTG).

This work makes the following contributions:

- We show that context-agnostic affine/convex gap penalties do not accurately reflect the likelihood of nanopore sequencing errors in *n*-polymer regions
- We extend Needleman-Wunsch affine gap alignment to include context-dependent gap penalties for more accurately aligning *n*-polymers
- We identify that during germline small variant calling, most INDEL false negative errors occur during the pileup-based variant calling stage
- We introduce “follow-banding” for efficient read realignment
- We develop a VCF standardization method that ensures variants are reported in the same format as our nPoRe realigner
- We show that haplotype phasing and nPoRe realignment significantly improve pileup-based variant calling accuracy

## Results

### Overview

This work focuses on improving the accuracy of germline small variant calling (heritable mutations $<50$ bp in size). We do so by realigning mapped reads (inputting and outputting in standard BAM format) to improve fine-grained alignment and read concordance by adjusting each read’s CIGAR string. Because we are concerned only with small variants, performing realignment within a $\pm \,50$ bp window of the original mapping/alignment is sufficient. This work is independent of downstream variant caller, and nPoRe can be used in combination with either Clair3 or PEPPER. To evaluate nPoRe, we retrain Clair3 from scratch with minimap2- and nPoRe-realigned reads. We find that when retraining Clair3, it is beneficial to “standardize” the ground truth VCF to report variants in a manner similar to nPoRe-realigned reads (details in "Methods" section). Realigning reads with nPoRe is relatively efficient and results in a significant increase in read concordance, which translates well to an improvement in final variant calling accuracy.

### Accuracy

**Fig. 6:**
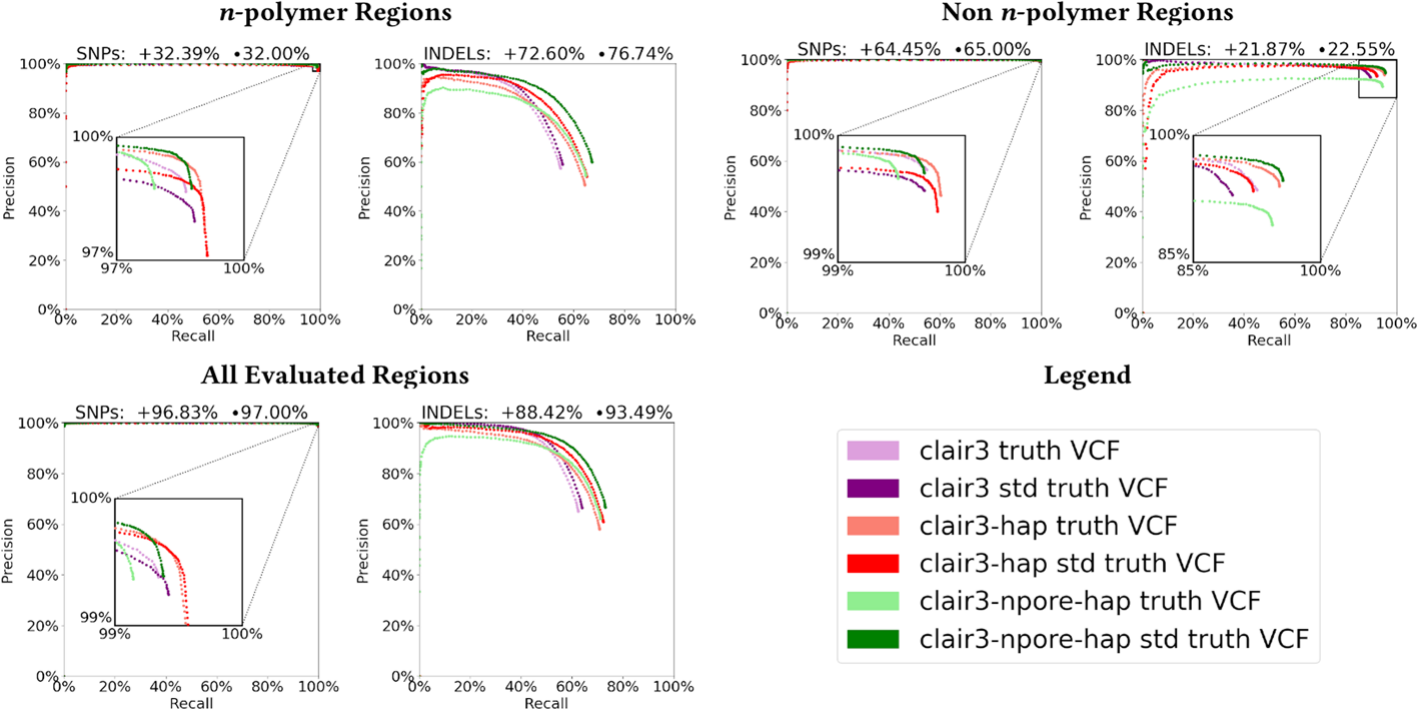
Accuracy results for each Clair3 pipeline stratified by region. Results are reported for both the original (lighter colors, denoted $+$) and standardized (darker colors, denoted $\bullet$) ground-truth VCFs, and titled with the percentage of SNP/INDELs within each region according to both truth VCFs

Figure 6 reports the performance of all three evaluated Clair3 pipelines, with precision and recall for SNPs and INDELs given separately for each sub-region. Results are reported for both the original and standardized ground-truth VCFs (see "Methods" section). Figure 6 shows that *n*-polymer regions are responsible for the majority of INDEL errors, since with these regions excluded, INDEL precision and recall both exceed $95\%$. Performance in tandem repeat regions alone is relatively good, and homopolymers account for the majority of remaining errors. For a fixed INDEL precision of 2/3, sorting reads by haplotype (clair3$\rightarrow$clair3-hap) improves INDEL recall from 63.76 to $70.66\%$. Realigning reads with nPoRe (clair3-hap$\rightarrow$clair3-npore-hap) further improves INDEL recall to $73.04\%$.

We chose to perform evaluations using both VCFs because although they contain the exact same information, the “standardized” VCF was more likely to report several INDELs instead of several SNPs (due to the lower *n*-polymer shortening/lengthening penalty), and occasionally broke an INDEL up into several smaller INDELs. As a result, the standardized VCF had $18.05\%$ more INDELs (31,104) and $1.45\%$ fewer SNPs (155,163) than the original VCF (25,500 INDELs and 157,454 SNPs). hap.py’s vcfeval engine assigned partial credit for SNPs less frequently to nPoRe-aligned reads. The standardized VCF resulted in apparent higher INDEL recall for all variant callers, due to the increase in total INDELs.

### Read concordance

**Fig. 7:**
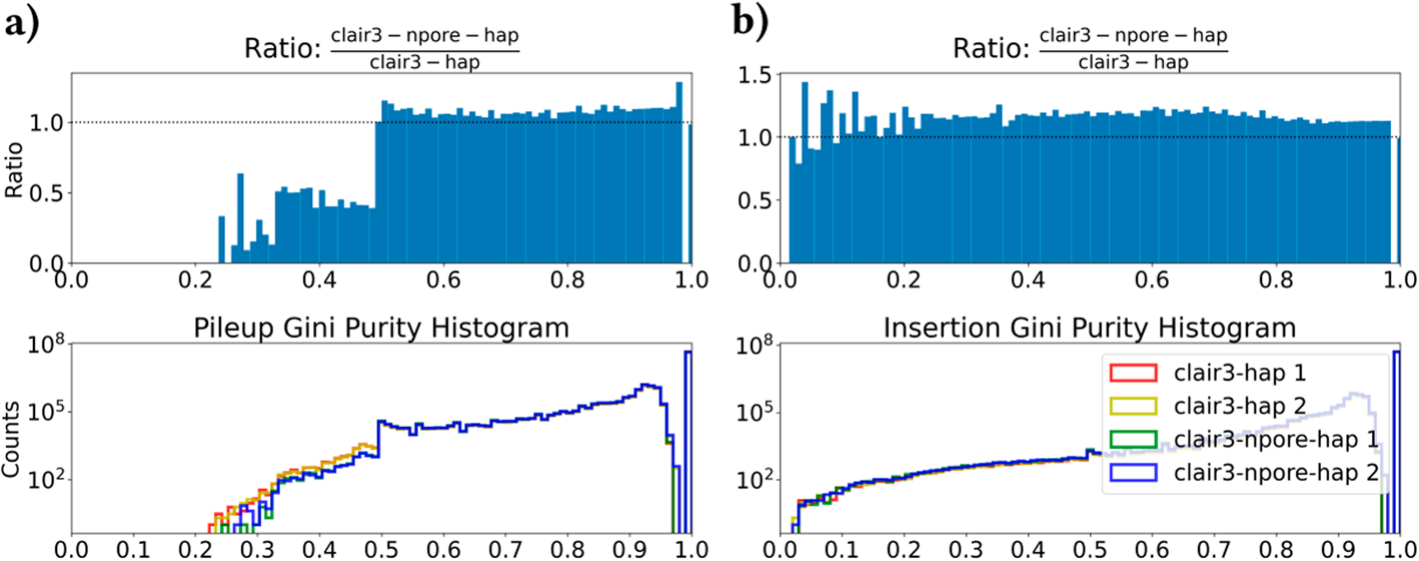
Read concordance: Gini purity histograms for **a** pileup columns and **b** insertions

If sequenced reads were to not contain errors, they would all perfectly agree with one another and variant calling would be easy. We would like to maximize the extent to which reads agree with one another, which we term “concordance” and measure per-haplotype and per-position in terms of Gini purity. Gini purity is defined as $GP = \sum _{i=1}^N \mathbb {P}(i)^2$, where *N* is the number of classes and $\mathbb {P}(i)$ is the probability of class *i*. Figure 7a (lower graph) shows the resulting Gini purity histogram with the classes A, C, G, T, - (deletion) on a logarithmic y-scale. If all reads agree, $GP=1$. If $50\%$ call C, $GP=0.5$. In the worst case, where there is an even split between the five classes, $GP=0.2$. Reference positions with low Gini purity scores are therefore difficult to call, and are a likely source of both false positive and false negative variants. The lower graph in Fig. 7 compares the Gini purity score distributions in minimap2 and nPoRealigned BAMs. It shows a marked $\approx 50\%$ decrease in positions with Gini purity less than 0.5 for the nPoRe-realigned BAM, demonstrating that nPoRe greatly improves alignment concordance across reads in difficult regions.

Read concordance in the phased BAM pileup, evaluated by Gini purity computed per reference position, is shown in Fig. 7a. Insertion concordancy was evaluated separately, in Fig. 7b, where the classes are all insertions between base *k* and $k+1$ (e.g. $\epsilon ,A,AA,AAA,AT,ATT...$). We plot insertions separately because the variable number of classes greatly affects the Gini purity score distribution. There appears to be an approximately $10\%$ increase for all imperfect Gini purity scores, which we attribute to nPoRe’s increased likelihood of calling INDELs (and as a result, more average classes and greater divergence).

### Timing

We performed our evaluations on a system with $2\times$ Intel Xeon E5 2697v3 2600MHz CPUs and 64GB total RAM. Timing results are shown in Table 1. From this evaluation, it is clear that any pipeline stages requiring computation on the full BAM file (marked with *) are considerably more expensive than working with just putative variants, a small fraction of the entire genome. Although our nPoRe realigner accounted for $79.6\%$ of total CPU time, it only accounted for $27.8\%$ of the real runtime, or just under twice as long as it took to index the BAM. nPoRe’s CPU time was $51.6\times$ its real time on our system with 56 total cores, demonstrating that we took full advantage of the available parallelism.

**Table 1:** Timing results for stages in the clair3-npore-hap pipeline (Table 3)

Step	Real time	CPU time
*Align reads	1218:01	7808:24
Generate tensors	44:29	301:53
Train clair3	38:18	54:22
Call variants	86:26	2392:17
Phase variants	347:58	344:05
*Phase reads	240:32	228:03
*Index BAM	1138:09	1750:13
Phase truth VCF	364:23	360:03
Standardize truth VCF	546:31	3756:46
*Realign reads	2168:15	111,792:55
*Generate haplotype tensors	1363:23	3162:48
Train model	41:40	66:29
Call variants	200:08	8343:01

Asterisks (*) denote steps for which the full BAM is required, rather than just the VCF

### Score matrices

**Fig. 8:**
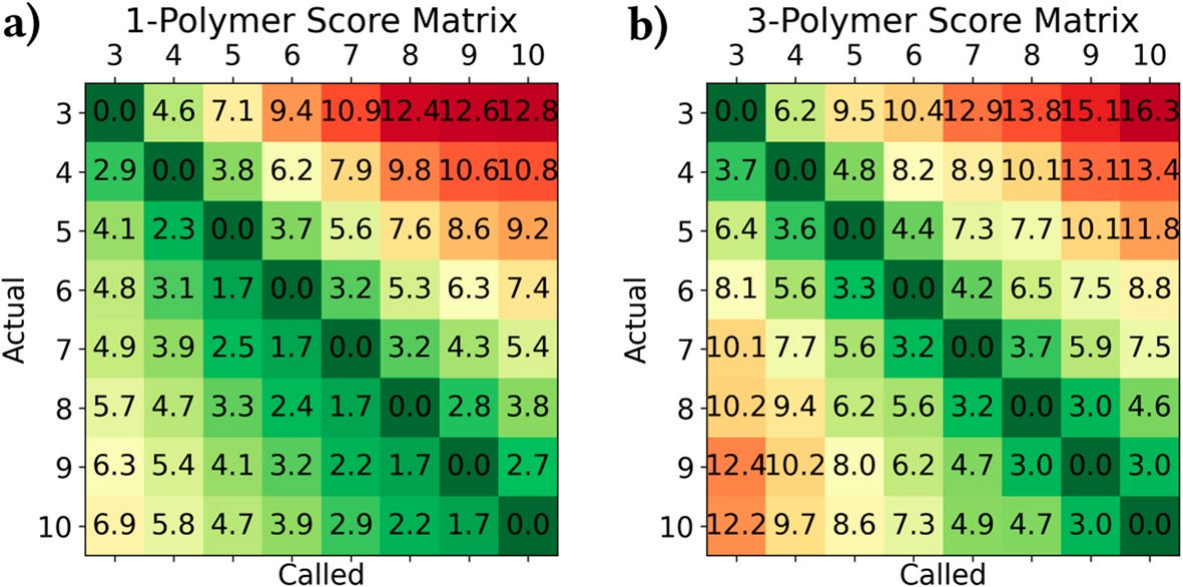
**a** 1-polymer and **b** 3-polymer score matrices. Scores are equivalent to the measured negative log probability of each error

Figure 8 shows the calculated score matrices for 1- and 3-polymers, corresponding to the confusion matrices in Fig. 2. In general, *n*-polymer INDELs are penalized less than the general-case affine gap INDEL penalty. Additionally, insertions are more common than deletions, and INDELs are more common in *n*-polymers of shorter repeat unit length (*n*).

## Discussion

The current nPoRe algorithm implementation was designed to demonstrate that there is a significant difference in INDEL rates between repetitive and non-repetitive sequences, due to the common occurrence of *n*-polymer copy number INDELs and sequencing errors. In order to do so, we decided upon a strict definition of *n*-polymers that requires at least three repetitions of the exact same repeat unit. We found that this strict definition includes around $65\%$ of all INDELs in our dataset. Despite this, there are many repetitive regions in which sequencing errors are common but do not meet our strict definition of an *n*-polymer. For example, the sequence AAATAAAATAAATAAAT is not an *n*-polymer because the second repetition of AAAT has an additional A. A more lenient definition of *n*-polymers would result in a broader application of reduced INDEL gap penalties for repetitive regions and may improve alignment results further. Additional leniency, however, would come at the cost of increased computation.

We find alignment speed to be the greatest practical limitation of our nPoRe aligner, despite writing our alignment kernel in Cython and taking full advantage of the available parallelism. Genomics datasets are inherently large, and a hyper-optimized implementation with SIMD intrinsics and reduced data width may be necessary for large-scale applications. We have already explored reducing memory usage by shifting to a difference-based *n*-polymer cost matrix and only storing $2*n_{\textrm{max}}+1$ matrix rows in memory, resulting in about 10–20 Replacing the *n*-polymer cost matrix with a best-fit surface or function would likely improve efficiency further by reducing irregular memory accesses. We consider the main contribution of this work to be identifying fine-grained alignment as a significant source of small variant calling INDEL errors and developing an algorithmic solution. Speed can be improved through further engineering efforts.

The astute reader may notice that our *n*-polymer copy number INDEL penalties were calculated based on the measured negative log likelihood of occurrence in the original BAM, which as we’ve pointed out, has issues with fine-grained alignment. Even if this were to affect our estimate of *n*-polymer copy number INDEL likelihoods by $2\times$, however, the effect on INDEL penalty is only $\log {2}\approx 0.69$. Our algorithm has already reduced the cost of a 3-base deletion within a 3-polymer from $G_{\textrm{open}} + 2*G_{\textrm{extend}}=7$ to the range [3.0, 3.7], depending on the 3-polymer length. If necessary, a second iteration of INDEL likelihood estimation using the nPoRe-realigned BAM could be used to further improve score estimation.

## Conclusions

We identify the main source of nanopore germline small variant calling errors to be copy number INDEL false negatives in *n*-polymer regions, and show that context-agnostic affine gap penalties do not accurately reflect the likelihood of nanopore sequencing errors. To improve nanopore pileup-based variant calling accuracy, we explore correcting fine-grained read alignment. This work extends Needleman-Wunsch affine gap alignment to include repeat-aware gap penalties for *n*-polymers. In doing so, we also develop “follow-banding” for efficient long read realignment and a method for standardizing ground-truth VCFs. We demonstrate that read realignment improves read concordance and variant calling accuracy, and release nPoRe^1^ as an open source tool.

Despite being located in low-complexity regions, calling the length of tandem repeats is clinically relevant. There is an entire class of neuropathological disorders associated with copy number variation known as “Tandem Repeat Disorders”, or TRDs. Huntington’s Disease is one such disorder caused by 40 or more repeats of the CAG 3-polymer at the end of the gene *HTT*, instead of a normal 10–30 copies. Other TRDs include Fragile X Syndrome, Kennedy’s Disease, mytonic dystrophy, and several spinocerebellar ataxias [33]. Since nPoRe improves significantly improves read alignment and variant calling in tandem repeat regions, it will lead directly to more accurate diagnoses of such disorders.

## Methods

### Overview

Because we have designed a read **realignment** algorithm, we trust the initial mapping of each read. Each read and its corresponding section of the reference genome are realigned, and a new traceback (alignment path) is computed. In other words, our solution simply adjusts the CIGAR string of each read within the input BAM file to better model the most likely mutations and sequencing errors in an effort to achieve greater concordancy between reads.

Our realignment algorithm is an extension of the Needleman-Wunsch algorithm for global alignment [34]. In addition to including known improvements such as an affine gap penalty and custom substitution penalty matrix [35], our algorithm allows the shortening and lengthening of homopolymers and tandem repeats (i.e. ACACAC$\rightarrow$ACACACAC).

### *n*-polymer repeats

The literature often categorizes sequences consisting of one repeated base as “homopolymers”, and repeated sequences of at least two bases as “tandem repeats” or “copolymers” [8, 36]. Short tandem repeats (STRs) are often defined as repeated units 2–6 bases in length, and are also known as “microsatellites” or “simple sequence repeats” (SSRs) [37]. Rather than treating these classifications separately for nPoRe, **we define an** ***n*** **-polymer to consist of at least 3 exact repeats of the same repeated sequence, where the repeat unit is of length 1–6 bases** ($1\le n \le 6, l \ge 3$). For example, homopolymers such as AAAAA ($n=1$) and tandem repeats such as ACACACAC ($n=2$) and TTGTTGTTG ($n=3$) are *n*-polymers. Shorter or irregular repeated sequences such as AATTAATT and ACAACAAACAC are not.

An upper threshold of $n_{\textrm{max}}=6$ was selected because there is a marked decrease in the frequency of *n*-polymers for $n>6$. Tandem repeats are usually defined with the same upper bound on repeat unit length *n* for the same reason. Figure 4 shows that 6-polymers are already uncommon. Furthermore, nanopore R9.4.1 sequencers fail to accurately call the length of *n*-polymers because the pore’s effective sensing width is 5–6 bases; i.e. the measured signal depends upon 5–6 adjacent bases simultaneously [38]. An *n*-polymer for $n \le 6$ is usually observed as a nearly-constant signal due to exact repetition, from which it is difficult to determine repeat length. For *n*-polymers where $n>6$, this is less of a problem, and fewer errors are observed. For a similar reason, a minimum *n*-polymer length of $l=3$ was decided upon to classify a repeated sequence as an *n*-polymer. If $l=2$, there is never a series of *n* bases bordered on both sides by another copy of the same *n* bases. The two copies of *n* bases are each adjacent to a non-repeating region, and as a result the measured signal is non-constant and few basecalling errors occur.

### Penalty functions

For each read, differences from the reference genome can be attributed to either sequencing errors or actual mutations. Regardless of origin (error or mutation), our goal is to align these reads to the reference in a manner that accurately captures the change that occurred. Existing aligners fail to do this by defining substitution and gap penalties based on estimated rather than measured rates of occurrence, and the algorithms do not account for common sequencing error modes such as tandem repeat length errors in nanopore sequencers. In contrast, we calculate penalties based on frequency measurements from the input BAM file. We define the penalty score for each difference (whether error or mutation) to be the negative log likelihood of that event occurring. As a result, finding the minimum-penalty alignment path is equivalent to finding the most likely set of errors and mutations that have occurred (assuming independence).

#### Substitution penalty matrix

**Fig. 9:**
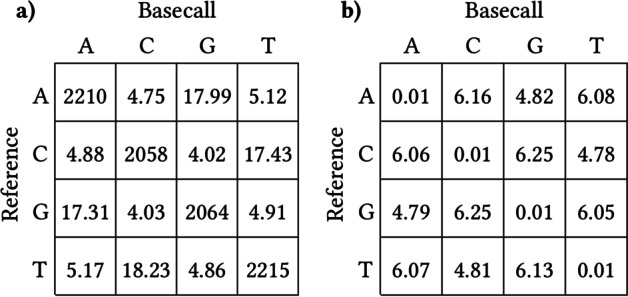
**a** substitution confusion matrix $C_P$, count in millions, and **b** resulting penalty matrix *P*

Figure 9 shows the calculation of substitution penalty matrix *P* from confusion matrix $C_P$, using Eq. 1. $\epsilon =0.01$ was included for numerical stability in the case that certain events were never observed. If we consider bases $x=``ACGT''$, then *P*[*i*, *j*] is the negative log probability that base *x*[*i*] was observed as base *x*[*j*], either through a mutation or sequencing error:

1 $$\begin{aligned} P[i,j] \approx -\log {\mathbb {P}(x[i]\rightarrow x[j])} \approx -\log {\frac{\mathrm {C_P[i,j]}+\epsilon }{\mathrm {sum(C_P[i,:])}+\epsilon }} \end{aligned}$$

#### Affine gap penalties

Confusion matrices for insertions ($C_I$) and deletions ($C_D$) were first generated by measuring the occurrence of small INDELs in the input BAM. Both matrices are 1D, since the expected INDEL length is always zero. Then, penalties were calculated by determining the negative log probability of each INDEL length *i* occurring: $-\log \frac{C_I[i]+\epsilon }{\textrm{sum}(C_I)+\epsilon }$. From these penalties, a best-fit gap opening penalty $G_{\textrm{open}}$ of 5 and gap extension penalty $G_{\textrm{extend}}$ of 1 was selected for both insertions and deletions [35].

#### Tandem repeat penalty matrix

First, confusion matrix $C_N$ of shape $6\times 100\times 100$ was generated by comparing expected and observed *n*-polymer lengths *l* (up to 100). For each *n*, or repeat unit size 1–6, a penalty matrix was calculated using the following equation, where *i* is the expected repeat length and *j* is the measured repeat length.

$$\begin{aligned} N[n,i,j] \approx -\log {\mathbb {P}(n,i,j)} \approx -\log {\frac{C_N[n,i,j]+\epsilon }{\textrm{sum}(C_N[n,i,:])+\epsilon }} \end{aligned}$$

To improve penalty regularity, particularly for longer *n*-polymers where few examples were observed, the following two properties were enforced for each possible combination of $k>0, n, i$ within the bounds of *N*:

- Shorter INDELs are more likely:
  $$\begin{aligned} N[n,i, i \pm k] > N[n,i, i\pm (k+1)] \end{aligned}$$
- Longer *n*-polymers are more likely to contain an INDEL of a given size:
  $$\begin{aligned} N[n,i+1,(i+1)\pm k] > N[n,i,i \pm k] \end{aligned}$$

### Reference annotation

Reference annotations are used to track eligible *n*-polymers during alignment. For each possible *n*-polymer repeat unit length from $n=1$ to $n_{\textrm{max}}$, each reference position is annotated with *l*, the length or number of consecutive repeat units, and *idx*, the 0-based index of the current repeat unit ($0\le idx < l$). Table 2 shows example annotations for a short reference sequence for $n=1$ and $n=2$. Recall that in order for a sub-sequence to be considered an *n*-polymer, the pattern must repeat exactly at least three times. Annotations may overlap, and non-zero annotations are only placed at the start of every *n*-polymer repeat unit.

**Table 2:** Example *n*-polymer reference annotations

Reference:		A	T	A	T	A	T	A	T	T	T	T	T	A	A	A	G	C	G	C	G	C
*n = 1*																						
l:		0	0	0	0	0	0	0	5	5	5	5	5	3	3	3	0	0	0	0	0	0
idx:		0	0	0	0	0	0	0	0	1	2	3	4	0	1	2	0	0	0	0	0	0
*n = 2*																						
l:		4	3	4	3	4	3	4	0	0	0	0	0	0	0	0	3	0	3	0	3	0
idx:		0	0	1	1	2	2	3	0	0	0	0	0	0	0	0	0	0	1	0	2	0

### Alignment

Before aligning read *r* to reference *R*, the reference is annotated with *n*-polymer information as discussed previously. Then, the five matrices *D*, *I*, *M*, *S*, and *L* are computed in lockstep one cell at a time, in that order. These matrices of size $|r|\times |R|$ represent the states Deleting, Inserting, Matching, Shortening *n*-polymers, and Lengthening *n*-polymers, respectively. For each cell, each matrix stores a tuple (*val*, *pred*, *run*) containing the accumulated penalty *val*ue, in addition to the *pred*ecessor matrix and consecutive movements (*run*) within that matrix for backtracking purposes.

**Fig. 10:**
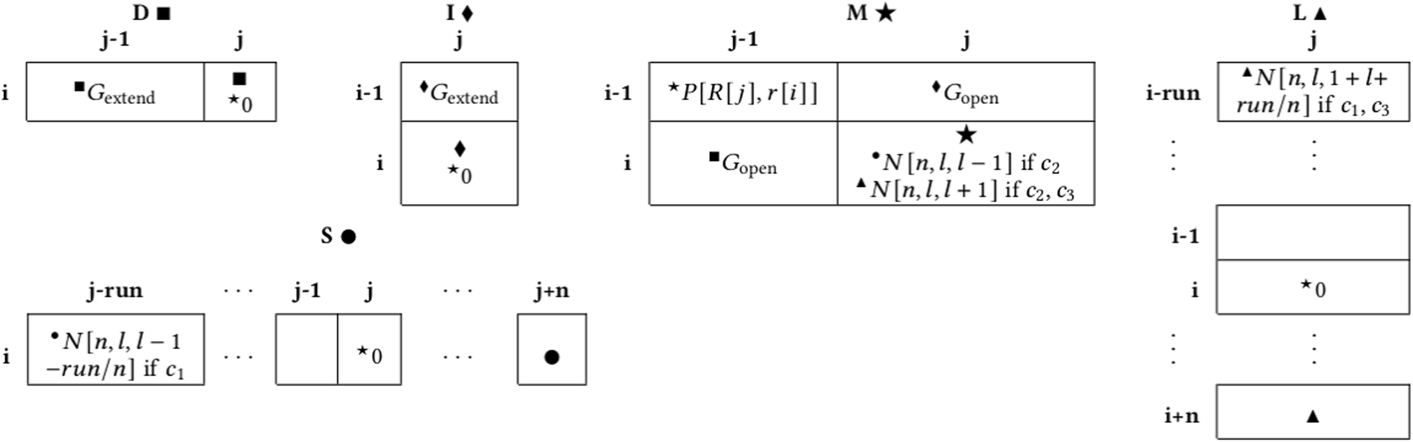
Realignment algorithm dependencies and penalties for computing cell *i*, *j* in the five matrices $D\blacksquare , I\blacklozenge , M\bigstar , S\bullet , L\blacktriangle$, which represent the states Deleting, Inserting, Matching, Shortening, and Lengthening, respectively. Computation for cell *i*, *j* occurs in that order, and large symbols (e.g. $\blacksquare$) denote where the result is stored. Superscript symbols (e.g. $^\blacksquare$) represent cell dependencies, and a penalty score accompanies it. Each result is the minimum value of all dependency cells plus their accompanying penalty scores. *n*-polymer shortening and lengthening is only allowed if certain conditions are met ($c_1, c_2, c_3$), described in the text

Figure 10 demonstrates the cell dependency patterns and penalties in greater detail. For example, when computing cell *i*, *j* in *S*, the reference annotations *l* and *idx*, are first retrieved for each *n* for *R*[*j*]. All dependencies (marked with $^\bullet$ for *S*) in Fig. 10 are considered, and the minimum value of these dependencies’ cell values plus the associated penalties is calculated and stored in the result cell (marked with $\bullet$ for *S*). In other words, when looking at *S*[*i*, *j*], for each *n*, we do:

$$\begin{aligned} S[i,j+n] = \min ( M[i,j] + N[n,l,l-1],\\ S[i,j-run] + N[n, l, l-1-run/n]) \end{aligned}$$

These two movements correspond to starting to shorten a tandem repeat (state $M{\rightarrow }S$), and continuing to shorten a tandem repeat (state $S{\rightarrow }S$). All movements into matrices *S* and *L* such as these are only allowed conditionally based on reference annotations (described in the following section). Note that if matrices $S\bullet$ and $L\blacktriangle$ are omitted (as well as all $^\bullet$ and $^\blacktriangle$ dependencies), this algorithm is equivalent to Needleman-Wunsch alignment with an affine gap penalty [34].

### *n*-polymer INDEL conditions

Unlike matrices *D*, *I*,  and *M*, the results for matrices *S* and *L* are stored several cells ahead of the current cell, and cell dependencies are only allowed conditionally based on the reference annotations. This ensures that matrices *S* and *L* only allow INDELs which change the copy number of tandem repeats and homopolymers. Here are the three conditions $c_1,c_2,c_3$ used by our algorithm, and referenced in Fig. 10:

$$\begin{array}{*{20}l} {c_{1} = } \hfill & {amp;l} \hfill & {gt;0} \hfill & {amp;{\text{start}}{\mkern 1mu} \;{\text{of}}\;{\mkern 1mu} {\text{repeat}}\;{\mkern 1mu} {\text{unit}}} \hfill \\ {c_{2} = } \hfill & {amp;l} \hfill & {gt;0\;{\mkern 1mu} and\;{\mkern 1mu} idx = = 0} \hfill & {amp;{\text{start}}{\mkern 1mu} \;{\text{of}}{\mkern 1mu} \;n{\mkern 1mu} \;{\text{ - polymer}}} \hfill \\ {c_{3} = } \hfill & {amp;R[j + 1:j + 1 + n] = = } \hfill & {amp;} \hfill & {} \hfill \\ {} \hfill & {amp;r[i + 1:i + 1 + n]} \hfill & {amp;{\text{next}}\;{\mkern 1mu} n{\mkern 1mu} \;{\text{bases}}\;{\mkern 1mu} {\text{of}}{\mkern 1mu} \;r{\mkern 1mu} {\text{match}}\;{\mkern 1mu} R} \hfill & {} \hfill \\ \end{array}$$

### Backtracking

**Fig. 11:**
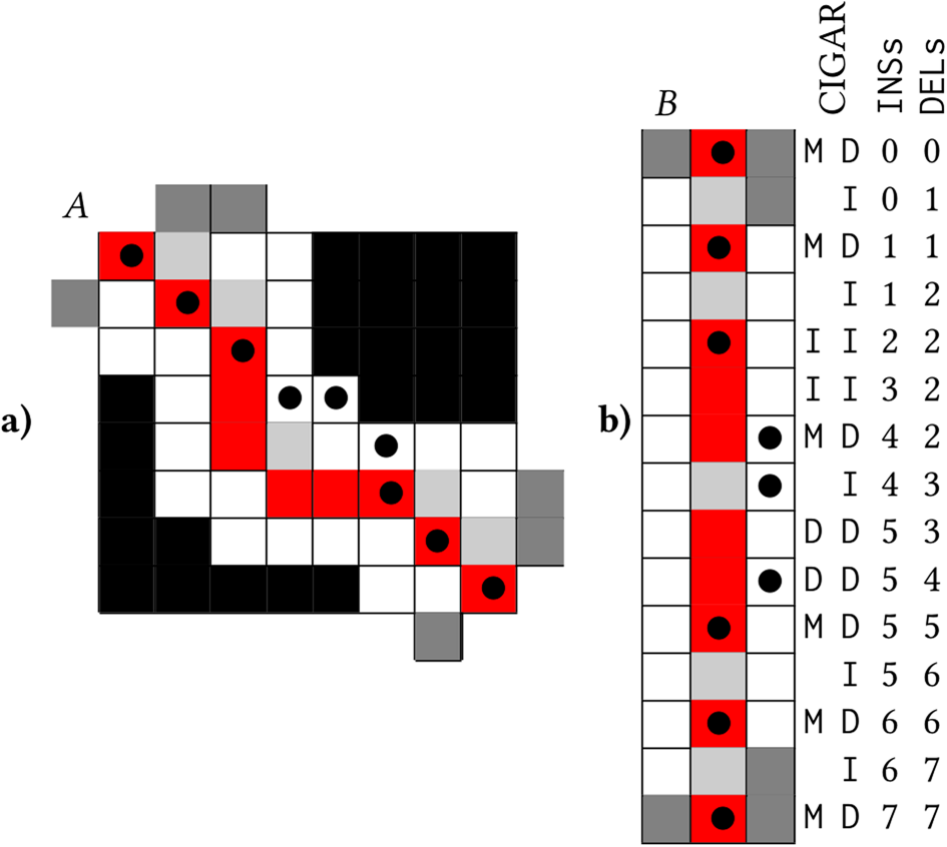
Follow banding matrix transformation $A \rightarrow B$. Red cells mark the original alignment path. Light gray cells mark supplements to the original alignment path, with diagonal Matches split into a horizontal Deletion plus vertical Insertion. $\bullet$ marks the new alignment path. Black and dark gray cells are not computed due to being out of band and out of bounds, respectively. Rows in *B* correspond to banded diagonals of *A*, centered on the supplemented alignment path

Traceback occurs entirely within matrix *M*, and relies on *pred*ecessor and *run* length information computed during the forward pass. The selected optimal alignment path is computed by Algorithm 1 and reported in the output BAM file in the form of a CIGAR string. CIGAR strings are composed of the symbols M, I, and D. Reference Matches, Insertions, and Deletions, correspond to diagonal, vertical, and horizontal movements in the alignment matrix, respectively. An example alignment is denoted by $\bullet$ in Fig. 11a. After computation, the computed CIGAR string is collapsed (MMMDMIMM$\rightarrow$3M1D1M1I2M).
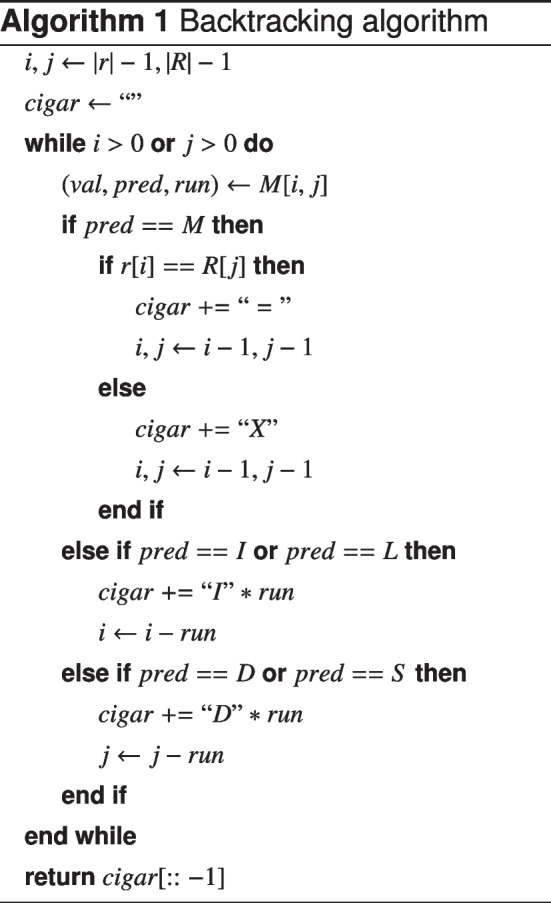


### Follow-banding

As mentioned previously, the primary goal of our read realignment algorithm is to more accurately model the mutations and sequencing errors in fine-grained alignment. Therefore, we can skip read mapping and trust the read start position reported by the previous aligner in the input SAM/BAM file’s POSition field. Read *r* will be aligned to a small subsection of the reference $R[\mathrm {\texttt {POS}}:\mathrm {\texttt {POS}}+m]$, where *m* is the length of the reference sequence corresponding to *r*. Barring any large INDELs, $m\approx |r|$, and we transform our alignment problem from *O*(|*R*||*r*|) to $O(|r|^2)$.

Moreover, we can use the SAM/BAM file’s existing CIGAR string to simplify our alignment problem even further. Our optimal alignment will likely follow a path close to that of the original alignment. Figure 11a demonstrates how we can compute the alignment matrix (new optimal path denoted by $\bullet$) in a narrow band $b=1$ that follows the original alignment (red cells). Dark gray and black cells are not computed. Essentially, we use the CIGAR string to precompute the movement directions for adaptive banded alignment as proposed by Suzuki and Kasahara [39], instead of using a heuristic comparing penalty scores on the band’s edge.

Firstly, all M CIGAR operations are converted to ID, an insertion followed by a deletion. This change is shown in Fig. 11, supplementing the original red alignment path with light gray cells. Next, computation proceeds one anti-diagonal row of width $2b+1$ at a time, centered on the alignment path. The computation of anti-diagonal rows shifts either right or downward at each step, governed by the previous CIGAR operation, D or I. These anti-diagonal rows can be stored efficiently in matrix format, as demonstrated in Fig. 11b. Transforming the banded $|r|\times |r|$ matrix *A* to a $(2b+1)\times 2|r|$ matrix *B* saves significant space because nanopore sequencing read lengths |*r*| can be up to several million bases [4], while realignment works well with a band width of $b=30$.

Offset arrays INSs and DELs can be precomputed using the CIGAR (Fig. 11). Given a cell in matrix *B* with indices *i*, *j*, its position in matrix *A* can be computed using the following formula:

$$\begin{aligned} \textrm{row} = \mathrm {\texttt {INSs}}[i] + b - j \textrm{col} = \mathrm {\texttt {DELs}}[i] - b + j \end{aligned}$$

### Time and space complexity

#### Reference annotations

The worst-case time complexity for computing the reference annotations is $O(|R|n_{\textrm{max}}^2l_{\textrm{max}})$, where |*R*| is the length of the reference *R*, and $n_{\textrm{max}}$ is the maximum *n*-polymer considered, and $l_{\textrm{max}}$ is the maximum *n*-polymer length. Since our *n*-polymer score matrix *N* is of size (6, 100, 100), $n_{\textrm{max}}=6$, and $l_{\textrm{max}}=100$. Thus, the time complexity is effectively *O*(|*R*|). Furthermore, these annotations must only be computed once, and cost can be amortized over all the reads that are aligned to the reference. We found the time required for reference annotations to be insignificant compared to alignment. These annotations require $O(|R|n_{\textrm{max}})$ space.

#### Read alignment

Once the reference annotations and score matrices have been computed, the nPoRe algorithm requires *O*(|*R*| |*r*|) time for each read *r*. The only additional overhead nPoRe incurs over Needleman-Wunsch with affine gaps is computing five Dynamic Programming (DP) matrices instead of three, as well as computing the new cell dependencies. All new penalties are conditional *O*(1) lookups. As discussed earlier, follow-banding further reduces both the time and space complexity of alignment from *O*(|*R*| |*r*|) to *O*(*b*|*r*|), where *b* is the band width. In total, the cost of aligning all reads is $O(\sum _{i=1}^m b\times |r_i|)$, where *m* is the number of reads, or equivalently *O*(*db*|*R*|), where *d* is the average depth of coverage.

All our code is open source and readily available at: https://github.com/TimD1/nPoRe.

### Datasets

#### Reference

We used the GrCh38 reference from the Genome-In-A-Bottle (GIAB) consortium [36].

#### Reads

We obtained our FASTQ files from *ONT Open Datasets’* May 2021 re-basecalling of HG002 PromethION R9.4.1 data using Guppy 5.0.6. Specifically, we used flow cell PAG07162, prepared using the Short Read Eliminator (SRE) protocol [40]. Depth of coverage was approximately $60\times$. For training, we used chr1-chr19, and for testing we used chr20-chr22.

#### Stratification regions

Stratification BED regions were calculated for $n=1...n_{\textrm{max}}$ using the definition of *n*-polymers provided previously. Regions were extended by a single base on each side (slop=1) to include variants occurring at the edges of *n*-polymer regions. These BEDS were then merged and complemented as necessary to create stratification BEDS for all *n*-polymer regions and non *n*-polymer regions.

### Pipeline

**Table 3:** Clair3 training and evaluation pipelines

**Step**	clair3	clair3-hap	clair3-npore-hap	Program
Align reads	$\checkmark$	$\checkmark$	$\checkmark$	minimap2
Generate tensors	$\checkmark$	$\checkmark$	$\checkmark$	clair3/CTP.py
Train clair3	$\checkmark$	$\checkmark$	$\checkmark$	clair3/Train.py
Call variants	$\checkmark$	$\checkmark$	$\checkmark$	clair3/run_clair3.sh
Phase variants		$\checkmark$	$\checkmark$	whatshap phase
Phase reads		$\checkmark$	$\checkmark$	whatshap haplotag
Phase truth VCF		$\checkmark$	$\checkmark$	whatshap phase
Standardize truth VCF			$\checkmark$	nPoRe/standardize_vcf.py
Realign reads			$\checkmark$	nPoRe/realign.py
Generate haplotype tensors		$\checkmark$	$\checkmark$	clair3-hap/CTPHaps.py
Train model		$\checkmark$	$\checkmark$	clair3-hap/Train.py
Call variants		$\checkmark$	$\checkmark$	clair3-hap/run_clair3.sh
Evaluate variants	$\checkmark$	$\checkmark$	$\checkmark$	hap.py

The full training and evaluation pipelines for all three Clair3 configurations tested are shown in Table 3. We used minimap2 version 2.17-r954-dirty [41], clair3 version v0.1-r9 [7], whatshap version 1.0 [42], and hap.py version v0.3.14 [36]. All three variant callers were trained from scratch using our $60\times$ HG002 dataset on minimap2-aligned reads for chr1-chr19, and tested on chr20-chr22. We first extended the retrained Clair3 baseline (clair3), phasing the input reads by haplotype and training a phased pileup candidate caller (clair3-hap). This was done because it significantly improves read concordancy, and leaving reads unphased when calling difficult single-haplotype variants might overshadow concordancy improvements gained by nPoRe’s alignment algorithm. This second baseline enables us the clearly delineate the gains from haplotype phasing and our nPoRe alignment algorithm. The final configuration was clair3-npore-hap, in which we performed ordinary variant calling with clair3, phased reads by haplotype, and then realigned them with nPoRe prior to variant calling.

### Haplotype phasing

In order to add haplotype phasing information to Clair3, a single iteration of the ordinary pileup-based pipeline was first run. Proposed variants were then phased using whatshap phase, and reads were tagged by haplotype using whatshap haplotag. We then sorted reads by haplotype into three separate BAM files. When generating the input pileup tensor for training clair3-hap and clair3-npore-hap, for each position a pileup tensor was generated for both unphased reads, reads from the first haplotype, and reads from the second haplotype. These three pileup tensors were then concatenated to create a new input tensor for Clair3.

### Truth VCF standardization

**Fig. 12:**
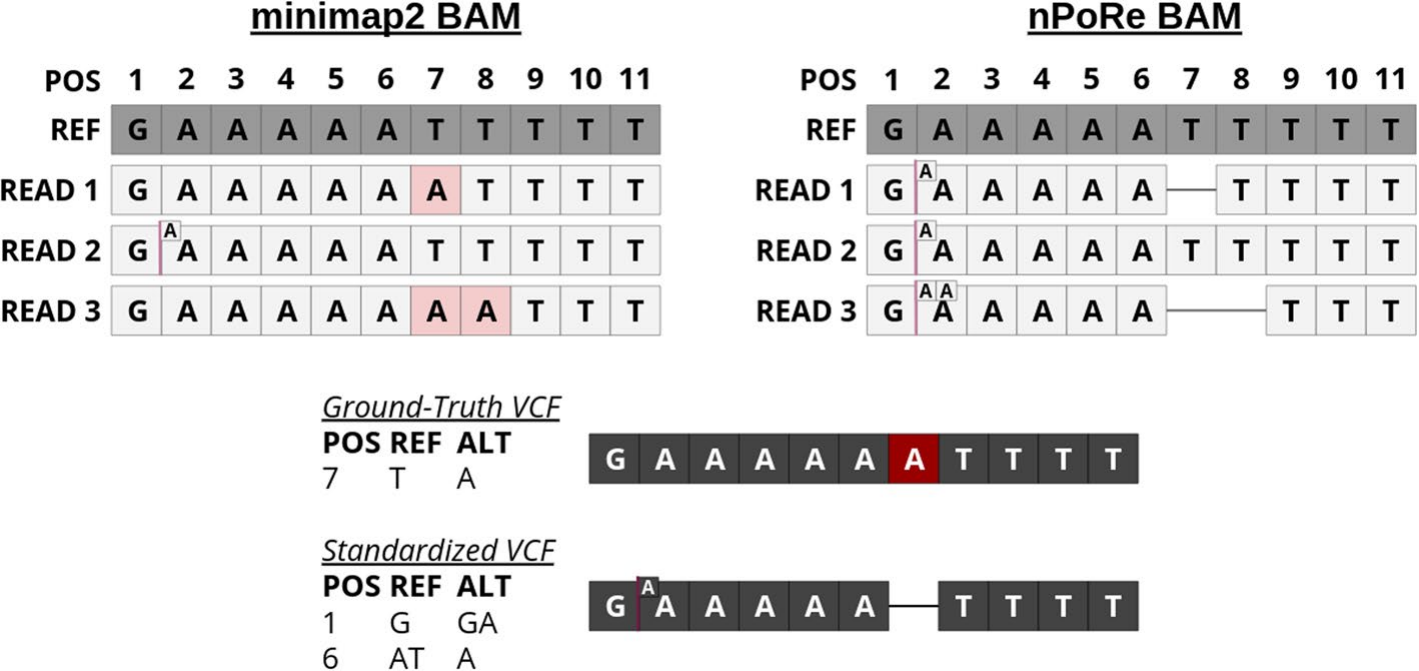
VCF Standardization: the ground-truth VCF is modified to report variants in a manner similar to nPoRe-realigned reads. The resulting sequence is unchanged

Figure 12 shows a simplified example of a typical minimap2 input BAM in comparison to the nPoRe-realigned output BAM. nPoRe is comparatively more likely to call *n*-polymer INDELs than SNPs, due to the reduced INDEL penalty. We found that clair3-npore-hap variant calling performance suffers if we train Clair3 with the original ground-truth VCF, since the realigned reads tend to report variants using an INDEL-heavy representation. To mitigate this, we altered the ground-truth VCF so that it reports variants using the same representation our aligner tends towards. An example of this “standardized” VCF is shown in Fig. 12.

To achieve this, we copied our reference FASTA to create two haplotype FASTAs, and applied the phased ground-truth variants to each haplotype FASTA, storing the new CIGAR. Using a mapping position of 0, the generated haplotype references, and associated CIGARs, we considered these ground-truth haplotype references to be reads and aligned them to the original reference using nPoRe. Any substitutions, insertions, or deletions in the resulting alignment were then parsed into a new standardized ground-truth VCF file. This process ensures that the new “standardized” truth VCF contains the same exact ground-truth sequence as the original VCF when applied to the reference FASTA, but reports variants in a manner consistent with nPoRe.

## Data Availability

The FASTQ dataset supporting the conclusions of this article is available in the ONT Open Datasets repository. For chromosomes N = 1 to 22, we downloaded: (s3://ont-open-data/gm24385_2020.11/analysis/r9.4.1/20201026_1644_2-E5-H5_PAG07162_d7f262d5/guppy_v4.0.11_r9.4.1_hac_prom/align_unfiltered/chrN/guppy_v5.0.6_r9.4.1_sup_prom/basecalls.fastq.gz). Specifically, we used flow cell PAG07162 from the May 2021 re-basecalling of HG002 PromethION R9.4.1 data using Guppy 5.0.6; more details regarding this data can be found here. We used the GrCh38 reference FASTA from the Genome-In-A-Bottle (GIAB) consortium [36], also made available through ONT Open Datasets: (s3://ont-open-data/gm24385_2020.09/config/ref/GCA_000001405.15_GRCh38_no_alt_analysis_set.fasta). The ground truth VCF and BED files were provided by GIAB at the following link: (https://ftp-trace.ncbi.nlm.nih.gov/ReferenceSamples/giab/release/AshkenazimTrio/HG002_NA24385_son/NISTv4.1/GRCh38/). The source code for nPoRe is available at (https://github.com/timd1/npore), and archived through Zenodo: (https://zenodo.org/record/6260902). nPoRe is written in Python/Cython and is available cross-platform through a public Docker container (https://hub.docker.com/r/timd1/npore) under the GNU GPLv3 license.

## Footnotes

1 https://github.com/TimD1/nPoRe.
